# Coordinated targeting of CK2 and KIT in gastrointestinal stromal tumours

**DOI:** 10.1038/s41416-019-0657-5

**Published:** 2019-11-28

**Authors:** Mengyuan Huang, Wenyu Yang, Jiaqing Zhu, Adrián Mariño-Enríquez, Chennianci Zhu, Jiaming Chen, Yuehong Wu, Yanping Quan, Haibo Qiu, Xuhui Li, Li Chai, Jonathan A. Fletcher, Wen-Bin Ou

**Affiliations:** 1 Zhejiang Provincial Key Laboratory of Silkworm Bioreactor and Biomedicine, College of Life Sciences and Medicine, Zhejiang Sci-Tech University, Hangzhou, China; 2 Department of Pathology, Brigham and Women’s Hospital and Harvard Medical School, Boston, MA USA; 3 State Key Laboratory of Oncology in South China, Sun Yat-sen University Cancer Center, Guangzhou, China; 4 Zhejiang Provincial Key Laboratory of Applied Enzymology, Yangtze Delta Region Institute of Tsinghua University, Jiaxing, Zhejiang China

**Keywords:** Cancer therapeutic resistance, Oncogenes

## Abstract

**Background:**

Most gastrointestinal stromal tumours (GIST) are driven by activating oncogenic mutations of KIT/PDGFRA, which provide a compelling therapeutic target. Our previous studies showed that CDC37, regulated by casein kinase 2 (CK2), is a crucial HSP90 cofactor for KIT oncogenic function and a promising and more selective therapeutic target in GIST.

**Methods:**

Biologic mechanisms of CK2-mediated CDC37 regulation were assessed in GISTs by immunoblotting, immunoprecipitations, knockdown and inactivation assays. The effects of a combination of KIT and CK2 inhibition were assessed by immunoblotting, cell viability, colony growth, cell cycle analysis, apoptosis, migration and invasiveness.

**Results:**

CK2 overexpression was demonstrated by immunoblotting in GIST cell lines and patient biopsies. Treatment with a specific CK2 inhibitor, CX4945, leads to CDC37 dephosphorylation and inhibits KIT signalling in imatinib-sensitive and in imatinib-resistant GIST cell lines. Immunoprecipitation demonstrated that CK2 inhibition blocks KIT:HSP90:CDC37 interaction in GIST cells. Coordinated inhibition of CK2 and KIT by CX4945 (or CK2 shRNA) and imatinib, respectively, leads to increased apoptosis, anti-proliferative effects and cell cycle arrest and decreased p-AKT and p-S6 expression, migration and invasiveness in all GIST cell lines compared with either intervention alone, indicating additive effects of inhibiting these two important regulators of GIST biology.

**Conclusion:**

Our findings suggest that combinatorial inhibition of CK2 and KIT warrants evaluation as a novel therapeutic strategy in GIST, especially in imatinib-resistant GIST.

## Background

Gastrointestinal stromal tumours (GIST) are the most common mesenchymal tumours of the digestive tract.^1–5^ The receptor tyrosine kinase (RTK) KIT (in ~80% of cases) and platelet-derived growth factor receptor alpha (PDGFRA, in ~5–10% of cases) mutant oncoproteins are crucial for GIST tumorigenesis and metastasis, as demonstrated by the clinical successes of small molecules targeting KIT and PDGFRA.^6–9^ RTK inhibitors imatinib, sunitinib and regorafenib are the standard first-, second- and third-line therapies, respectively, in patients with inoperable GIST,^10–12^ and adjuvant imatinib is used in patients with localised GIST with a high risk of recurrence.^13^ Most patients eventually experience clinical progression due to multiple imatinib-resistant mechanisms, which include acquisition of secondary mutations in the KIT kinase domain,^4,14^ genomic amplification of *KIT*, activation of alternate RTKs^15,16^ and loss of KIT expression.^3,17–19^

Various studies suggest that casein kinase 2 (CK2) regulates cell proliferation and metastasis in human cancers; there is increasing evidence of upregulation and activation of CK2 in non-small-cell lung cancer, breast cancer, colon cancer, prostate cancer and glioblastoma, through various pro-oncogenic mechanisms.^20–23^ CX4945 is a bioavailable small-molecule ATP-competitive inhibitor that targets CK2 active site. In preclinical models, CX4945 exhibits antitumour efficacy in breast cancer, prostate cancer and lung cancer,^24–26^ with synergistic effects in combination with chemotherapeutics, such as cisplatin or gemcitabine.^27,28^

Preclinical validations have shown compelling responses to inhibition of heat-shock protein 90 (HSP90) in GIST;^29–31^ however, targeting HSP90 has been challenging in clinical translation,^32,33^ due to the toxicity resulting from concomitant on-target inactivation of various other HSP90 client proteins, beyond KIT and PDGFRA. Protein kinases are the most prominent group of HSP90 clients and are recruited to the molecular chaperone by the kinase-specific cochaperone CDC37 (cell division cycle 37).^34^ Our previous studies have shown that CDC37 is a crucial HSP90 cofactor for KIT oncogenic expression in GIST.^35^ Opportunities for more selective HSP90 targeting might result from pharmacologic dysregulation of CDC37, in particular by modulation of CDC37 activation by phosphorylation at the Ser 13 residue. Because CK2 mediates the interaction of CDC37 and HSP90 through regulation of CDC37 Ser 13 phosphorylation,^36–38^ we hypothesised that CK2 inhibition could exhibit anti-proliferative and pro-apoptotic effects in GISTs, through CDC37 dephosphorylation and reduced CDC37:HSP90 interaction, resulting in inactivation of KIT and downstream intermediates. Coordinated targeting of CK2 and KIT in GISTs could exhibit strong anti-proliferative and pro-apoptotic effects in GISTs. In this study, overexpression of CK2 and phosphorylation of CDC37 at Ser 13 were demonstrated in GIST cell lines and GIST patient biopsies. Additive anti-proliferative/cytotoxic and pro-apoptotic effects were observed after combined inhibition of CK2 and KIT in GIST cell lines. CK2 inactivation increased imatinib sensitivity in drug-resistant GIST cells, indicating that combinatorial inhibition of the CK2 and KIT signalling pathway is a rational therapeutic strategy in GISTs, especially in imatinib-resistant GISTs.

## Methods

GIST cell lines and a number of the methods described below have been described previously.^15^

### Antibodies and reagents

Polyclonal antibodies to KIT (western), MAPK, GAPDH and HSP90 were purchased from Dako (Carpinteria, CA), Life Technologies (Carlsbad, CA), Proteintech Group Inc. (Rosemont, IL) and Santa Cruz Biotechnology (Santa Cruz, CA), respectively. All phospho-antibodies, polyclonal antibodies to AKT and the monoclonal antibody to S6 were purchased from Cell Signaling Technology (Beverly, MA). Monoclonal mouse antibodies to KIT (co-IP) and CK2 (Santa Cruz Biotechnology, CA), CDC37 (Abcam Biotechnology, Cambridge, MA) and β-actin (Sigma-Aldrich, St. Louis, MO) were used. Anti-mouse normal IgG was purchased from Cell Signaling Biotechnology. CX4945 (CX) and imatinib (IM) were purchased from Selleck (Houston, TX) and LC Laboratories (Woburn, MA, USA), respectively. Lentiviral CK2 shRNA constructs were purchased from The RNAi Consortium (TRC, Cambridge, MA, USA). Crystal violet and propidium iodide solution were purchased from Sigma-Aldrich. Protein A, Protein G beads, Lipofectamine and Plus reagent were purchased from Invitrogen (LIFE Technologies, USA). Puromycin and polybrene were purchased from Sigma (St. Louis, MO, USA).

### Cell lines

GIST882 is a human cell line established from an untreated GIST with a primary homozygous missense mutation in *KIT* exon 13, encoding a K642E mutant KIT oncoprotein.^39^ GIST-T1 was provided by Dr Takahiro Taguchi (Kochi University, Japan). GIST430/654 and GIST48 are human cell lines established from GISTs that had progressed on imatinib therapy, after initial clinical response. GIST430/654 has a heterozygous primary mutation in *KIT* exon 11, accompanied by a secondary exon 13 missense mutation (V654A). GIST48 has a homozygous *KIT* exon 11 mutation (V560D) and a heterozygous *KIT* exon 17 mutation (D820A).^40^ GIST cell lines (GIST430/654, GIST48 and GIST882), mesothelioma cell lines (MESO924 and MESO428) and liposarcoma cell lines (LPS141 and LPS510) were developed in Dr Fletcher’s Laboratory of the Department of Pathology at Brigham and Women’s Hospital. Ovarian cancer cell lines (SKOV3, OVCA429 and ES2) and non-small-cell lung cancer cell lines (PC-9 and A549) are kind gifts from Dr Ross Berkowitz and Dr Li Chai at Brigham and Women’s Hospital, respectively.

### Lentiviral CK2 shRNA constructs

Lentivirus preparations were produced by co-transfecting pLKO.1puro (empty vector or containing CK2 shRNAs), and helper virus packaging plasmids pCMV∆R8.91 and VSVG (at a 10:10:1 ratio) into 293T cells. Transfections were carried out using Lipofectamine and Plus reagent. Lentiviruses were harvested at 24, 36, 48 and 60 h post transfection, and frozen at −80 °C in aliquots of appropriate amounts for single-use infection. Well-validated shRNA was used for CK2 knockdown.

### GIST cell culture and virus infection

GIST-T1 and GIST882 cell lines were maintained in RPMI 1640 medium with 15% foetal bovine serum (FBS) supplemented with penicillin/streptomycin and 1% (v/v) l-glutamine. GIST430/654 and GIST48 cell lines were maintained in DMEM/F-12 with 15% FBS supplemented with penicillin/streptomycin and 1% (v/v) l-glutamine. GIST430 cells were seeded in six-well plates. Infections were carried out in the presence of 8 mg/mL polybrene. Following transduction, GIST430 was treated with 2 mg/mL puromycin to select for stable expression of the CK2 shRNA.

### Frozen tumour specimens

All GIST-frozen tumour specimens were analysed histologically and shown to be composed of >90% neoplastic cells. The studies were conducted in accordance with recognised ethical guidelines (U.S. Common Rule), and were approved by Brigham and Women’s Hospital and Sun Yat-sen University Cancer Center Institutional Review Boards under a discarded tissue protocol.

### Protein lysate preparations and western blot

Immunoblotting was performed after 6 h of treatment with CX4945 or imatinib in serum-free medium. Whole-cell lysates from cell lines were prepared using lysis buffer (IP buffer) (1% NP-40, 50 mM Tris-HCl, pH 8.0, 100 mM sodium fluoride, 30 mM sodium pyrophosphate, 2 mM sodium molybdate, 5 mM EDTA and 2 mM sodium orthovanadate) containing protease inhibitors (10 μg/mL aprotinin, 10 μg/mL leupeptin and 1 mM phenylmethylsulfonyl fluoride). Frozen tumour samples were diced into small pieces in cold lysis buffer on ice and homogenised with a Tissue Tearor (Model 398, Biospec Products Inc.) for 3 s, 3–5 times, on ice, and then rocked overnight at 4 °C. Lysates were cleared by centrifugation at 14,000 rpm for 30 min at 4 °C, and supernatant protein concentrations were determined using a Bio-Rad protein assay (Bio-Rad Laboratories, Hercules, CA). Electrophoresis and western blotting were performed as described previously.^41^ The hybridisation signals were detected by chemiluminescence (Immobilon^TM^ Western, Millipore Corporation, MA), and captured using an ImageQuant LAS4000.

### Immunoprecipitation

Sepharose-protein G beads with mouse monoclonal antibody were used. Immunoprecipitation was performed after 6 h of treatment with CX4945 (5 μM) in serum-free medium. In total, 1 mg of protein lysate was pre-absorbed for 30 min using 20 μl of protein G at 4 °C. Then, 2 μg of primary antibody against KIT or CDC37 was added to each supernatant and rocked for 2 h at 4 °C. In total, 20 μL of protein G beads were added and rocked overnight at 4 °C. The lysates were then spun at 10,000 rpm for 2 min at 4 °C, and beads were washed three times with 750 μL of IP buffer for 25 min followed once by 750 μL of 10 mM Tris-Cl buffer (pH 7.6). In all, 20 μL of loading buffer was added to the beads and boiled for 5 min at 95 °C. The interactions of KIT–HSP90–CDC37 were evaluated by specific antibody immunoblotting.

### Cell viability and apoptosis analysis

GIST-T1 (5000 cells/well) and GIST882, GIST430/654 and GIST48 cells were plated at 20,000/well in a 96-well flat-bottom plate (Falcon, Lincoln, NJ) and cultured in the RPMI 1640 or DMEM/F-12 for 24 h before treatment with CX4945 or imatinib. Proliferation studies were performed 3 or 6 days after inhibitor treatment in GIST cell lines (GIST-T1, GIST882, GIST430/654 and GSIT48) or in CK2-silenced GIST430 with stable shRNA expression using the CellTiter-Glo luminescence assay from Promega (Madison, WI). Viability was quantified using a Veritas™ Microplate Luminometer from Turner Biosystems (Sunnyvale, CA). Data were normalised to the control group (DMSO/pLKO). All assays were performed in quadruplicate wells, and were averaged from two independent experimental set-ups for each cell line.

Apoptosis was evaluated using the PE Annexin V Apoptosis Detection Kit I (BD Pharmingen, San Jose, CA). Briefly, GIST cells in six-well plates were treated with CX4945 (5 µM) or imatinib (0.05 and 0.5 µM for GIST-T1 and GIST882; 0.5 and 1 µM for GIST430/654 and GIST48) for 72 h, trypsinised and washed twice with cold PBS buffer and then treated with 5 µl of PE Annexin V and 5 µl of 7-AAD in 1× binding buffer for 15 min at RT (25 °C) in the dark. The stained cells were analysed in a flow cytometer (BD FACS Aria, Special Order System) within 1 h. CellQuest software (BD Biosciences) was used to analyse the data.

### Cell cycle analysis

GIST-T1, GIST882, GIST430/654 and GIST48 cells in six-well plates were trypsinised and washed with PBS buffer at 72 h after treatment with imatinib (0.05 and 0.5 µM for GIST-T1 and GIST882; 0.5 and 1 µM for GIST430/654 and GIST48) or CX4945 (2.5 and 5 µM). Nuclear staining was performed with a propidium iodide solution, and the cell suspension was analysed in a flow cytometer (FACScan, BD Biosciences) within 48 h. Data analysis was performed using ModFit LT (Macintosh).

### Colony-formation assay

Colony-formation assays were performed as published previously with minor modifications.^42^ In brief, GIST-T1 and GIST430/654 cells were plated at 10,000 cells/well in six-well plates and cultured in the RPMI 1640 or DMEM/F-12 for 4 or 14 days before treatment with imatinib (0.05 μM for GIST-T1 or 0.5 μM for GIST430/654) or CX4945 (2.5 and 5 μM). After treatment with inhibitors for 4 (GIST-T1) or 7 days (GIST430/654), the medium was removed, the cells were washed with PBS and they were then stained with 0.5% crystal violet in methanol for 20 min. Excess stain was removed by washing with distilled water. Colonies were photographed and counted. The experiments were performed in duplicate wells and repeated three times.

### In vitro wound-healing assays

Wound-healing studies were carried out as previously described.^43^ Briefly, slashes were created in near-confluent cell cultures using the tip of a P-100 pipetman after addition of inhibitors (imatinib or CX4945). Plates were photographed at days 0, 3, 4 and 8 using a Leica DMI 3000B inverted microscope (Leica Microsystems, Germany). Experiments were performed in triplicate.

### Cell migration and invasion assays

Migration and invasiveness of GIST cells were evaluated by the Matrigel assay (Collaborative Research Inc., Boston, MA), as previously described.^44^ Briefly, GIST cells (4 × 10^4^) were treated with imatinib (0.05 and 0.5 μM for GIST882, 0.5 and 1.0 μM for GIST430/654) or CX4945 (5 μM), followed by suspension in 0.5 ml of 0.5% serum-containing RPMI 1640/F-12 and seeded on the upper chamber of each well with 1.5 ml of 15% serum-containing medium added to the lower chamber, the higher serum content in the lower chamber providing a chemotactic gradient. After 72 h, noninvasive cells that remained on the upper surface of the filter were removed using a cotton swab, and cells that remained adherent to the underside of the membrane were fixed in 4% formaldehyde and stained with 0.1% crystal violet. Invasive cells were quantified in five contiguous fields of a fluorescence microscope, using a ×20 objective to obtain a representative number of cells. Experiments were performed in triplicate.

### Statistical analysis

Student’s *t* tests were performed on data from cells treated with inhibitors/shRNAs or DMSO/pLKO (control). Statistically significant differences between control and treatment were defined as *p < 0.05, **p < 0.01, ***p < 0.001 and ****p < 0.0001.

## Results

### CK2 overexpression in GIST cells

**Fig. 1:**
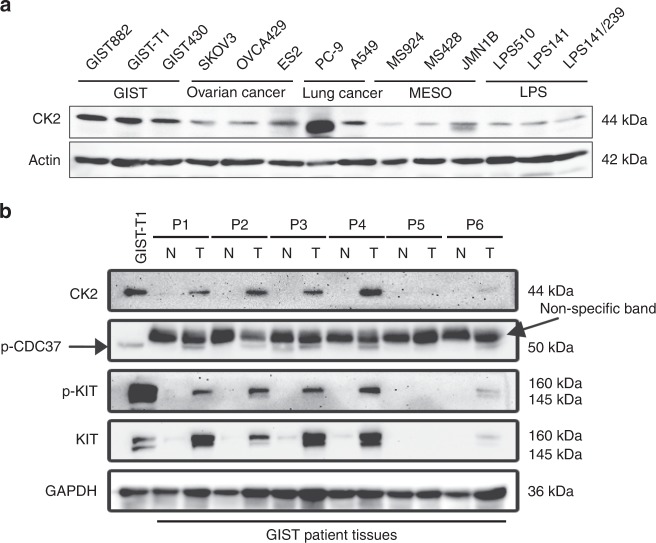
Immunoblotting evaluation of CK2 and phospho-CDC37 expression in GIST cell lines and GIST-frozen tumours. **a** CK2 expression levels in GIST cell lines (GIST-T1, GIST882 and GIST430/654) as compared with ovarian cancer (SKOV3, OVCA429 and ES2), non-small-cell lung cancer (PC-9, A549), mesothelioma (MESO924, MESO428 and JMN1B) and liposarcoma (LPS510, LPS141 and LPS141/239) cell lines. Actin stain is a loading control. **b** Expression of CK2, p-CDC37, KIT and activated p-KIT in GIST biopsies and adjacent non-neoplastic tissue samples. GAPDH stain is a loading control. Note the presence of a presumably cross-reacting, nonspecific band with the p-CDC37 antibody on tissue samples. Immunoblotting experiments were performed from two independent experiments for each sample.

CK2 expression was evaluated by immunoblotting in 14 patient-derived cell lines representing a wide range of human cancers: three GIST cell lines (GIST882, GIST-T1 and GIST430/654), three ovarian cancer cell lines (SKOV3, OVCA429 and ES2), two non-small-cell lung cancers (PC-9 and A549), three mesothelioma cell lines (MESO924, MESO428 and JMN1B) and three liposarcoma cell lines (LPS510, LPS141 and LPS141/239). The highest level of CK2 expression was detected in all three GIST cell lines, and in the EGFR-mutant NSCLC cell line PC-9 (Fig. 1a). Overexpression of CK2, phosphorylation of CDC37 and activation of KIT were demonstrated in five of six GIST biopsy samples (P1, P2, P3, P4 and P6), as compared with adjacent normal tissues. A single GIST biopsy sample P5 with undetectable expression of CK2 was deemed uninformative, due to lack of expression of KIT, p-KIT and CDC37, which suggest low tumour content (Fig. 1b).

### CK2 inhibition inactivates KIT/PI3K/AKT/mTOR signalling via CDC37 dephosphorylation in GISTs

**Fig. 2:**
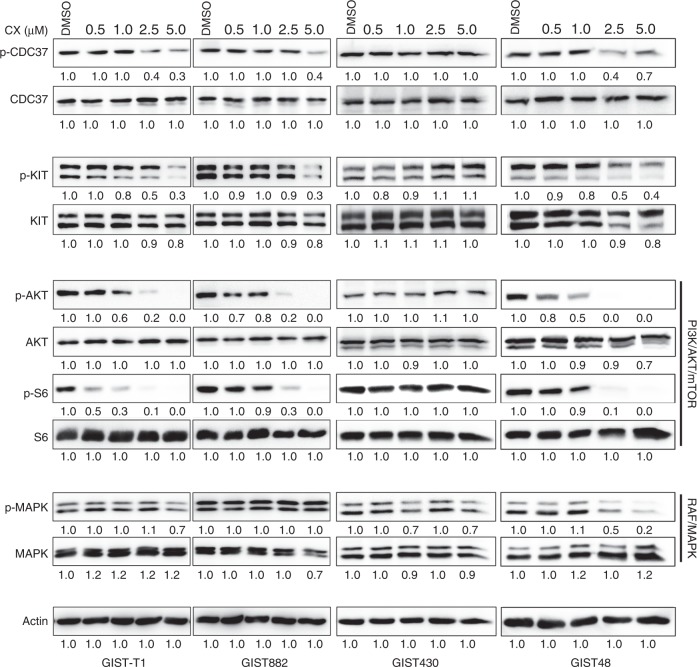
Inhibition of CK2 and KIT signalling intermediates in GIST cell lines after treatment with the CK2 inhibitor CX4945. Immunoblot evaluations of p-CDC37, CDC37, p-KIT, KIT, p-AKT, AKT, p-MAPK, MAPK, p-S6 and S6 in GIST-T1, GIST882, GIST430/654 and GIST48 after 6 h of CX4945 treatment in serum-free medium. Actin stain is a loading control. Immunoblotting experiments were performed from two independent experiments for each cell line. Linear capture quantitation of immunoblotting chemiluminescence signals, using an ImageQuant LAS4000. Intensity values are standardised to the DMSO control.

Given that the phosphorylation level of CDC37 at Ser 13 reflects CK2 activity,^45^ CDC37 and KIT/PI3K/AKT/mTOR signalling inhibition was evaluated by phospho-immunoblot analysis of total cell lysates in imatinib-sensitive cell lines (GIST-T1 and GIST882) and imatinib-resistant cell lines (GIST430/654 and GIST48) after 6 h of treatment with CX4945 (a specific inhibitor of CK2) in serum-free conditions (Fig. 2). KIT signalling intermediates were interrogated by immunoblotting, including PI3K/AKT/mTOR (S6), and RAF/MAPK after dephosphorylation of CDC37 by CK2 inhibition. CX4945 treatment led to a decrease in phosphorylated CDC37 and KIT in GIST-T1, GIST882 and GIST48, and inactivated downstream signalling intermediates AKT and S6 in a dose-dependent manner. However, CX4945 treatment had less effect on levels of p-CDC37, p-KIT, p-AKT and p-S6 in GIST430/654, at the doses of CX4945 tested. CX4945 treatment had little impact on CK2 expression in GIST-T1 and GIST882 (Supplementary Fig. 1), and MAPK activation in GIST-T1, GIST882 and GIST430/654, but decreased p-MAPK in GIST48 (Fig. 2). Signalling protein expression quantitations are shown in Fig. 2.

### Additive anti-proliferative effects via dual targeting of KIT and CK2 in GIST

**Fig. 3:**
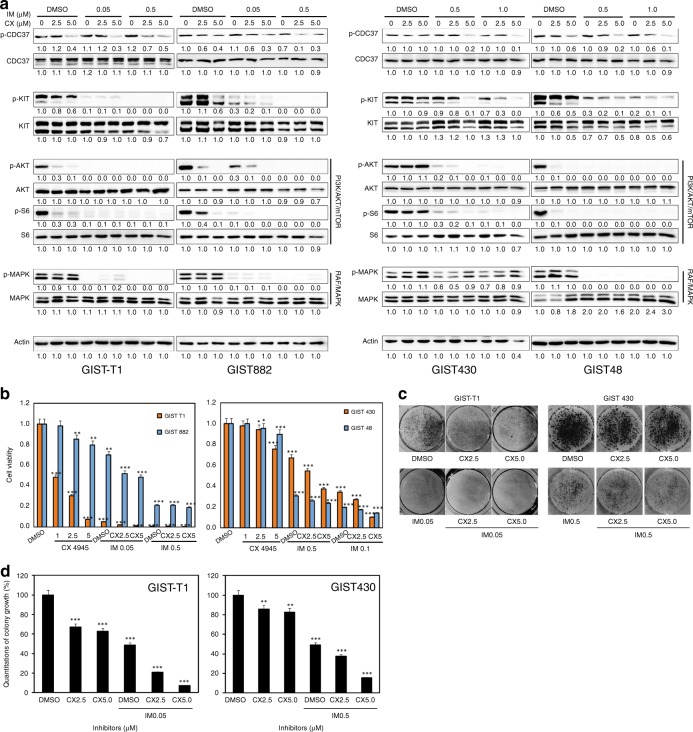

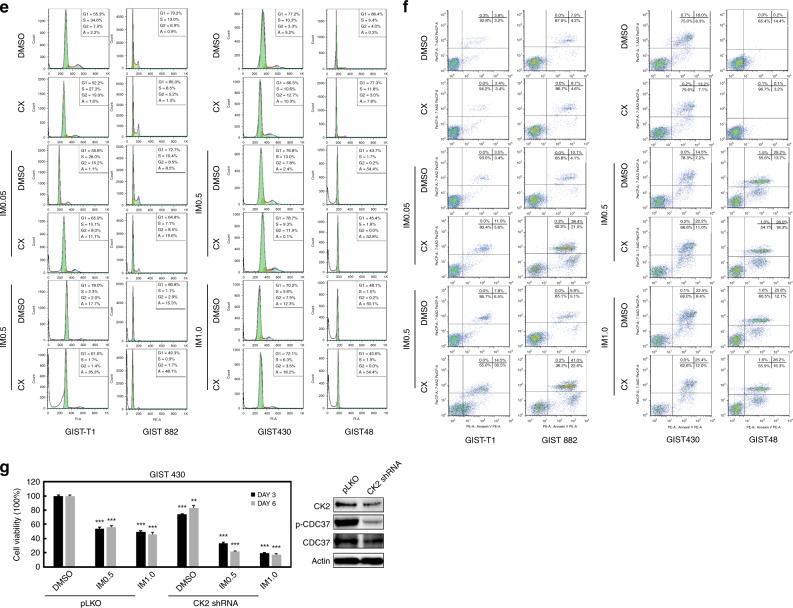
Additive effects of coordinated inhibition of KIT and CK2 as demonstrated by immunoblotting (**a**), cell viability (**b** and **g**), colony growth (**c**, **d**), cell cycle (**e**) and apoptosis assays (**f**), showing that combined inhibition of KIT and CK2 further decreases p-KIT, p-AKT and p-S6 expression, inducing greater anti-proliferative and apoptotic effects and cell cycle arrest, in GIST cell lines (GIST-T1, GIST882, GIST430/654 and GIST48), as compared with either intervention alone. **a** The CK2/KIT signalling (CDC37 and KIT) and downstream intermediates (AKT, MAPK and S6) were evaluated by immunoblotting at 6 h after treatment with CX4945 (CX: 2.5 and 5 µM) and imatinib (IM: 0.05, 0.5 and 1 µM). Actin stain is a loading control. Linear capture quantitation of immunoblotting chemiluminescence signals, using an ImageQuant LAS4000. Intensity values are standardised to the DMSO control. Immunoblotting experiments were performed from two independent experiments for each cell line. **b** Cell viability was evaluated by a Cell-titer Glo^®^ ATP-based luminescence assay in GIST cell lines (GIST-T1, GIST882, GIST430/654 and GIST48), 6 days after treatment with CX4945 (1, 2.5 and 5 µM) and IM (0.05, 0.5 and 1 µM). Data were normalised to DMSO and represent the mean values (±s.d.) from quadruplicate cultures, and were averaged from two independent experiments for each cell line. Statistically significant differences between untreated control and inhibitor treatments are presented as *p < 0.05, **p < 0.01, ***p < 0.001. **c** Colony growth assays were performed 4 and 7 days after treatment with CX4945 (2.5 and 5 µM) and IM (0.05 and 0.5 µM) in GIST-T1 and GIST430/654. Colony growth experiments were performed in triplicate. Combined KIT and CK2 inhibition led to a greater reduction in colony formation and size in GIST-T1 and GIST430/654 than either intervention alone. **d** Quantitation of GIST-T1 and GIST430/654 cell colony growth after treatment with IM and CX4945 for 72 h. Statistically significant differences between untreated control and inhibitor treatments are presented as **p < 0.01, ***p < 0.001. **e** Cell cycle analysis was performed 72 h after treatment with CX4945 (5 µM) and IM (0.05, 0.5 and 1 µM). GIST-T1, GIST882 and GIST48 showed substantial nuclear fragmentation after combination inhibition of KIT and CK2, and KIT inhibition alone. GIST430/654 showed substantial nuclear fragmentation after combination inhibition of KIT and CK2. Cell cycle experiments were performed in triplicate. **f** Apoptosis assays following IM (0.05, 0.5 and 1 µM) or/and CX4945 treatment for 72 h were performed with the PE Annexin V Apoptosis Detection Kit I. Apoptosis experiments were performed in triplicate. **g** Left, cell viability was evaluated by a CellTiter-Glo ATP-based luminescence assay in CK2 that stably silenced GIST430, 3 and 6 days after treatment with imatinib. Data were normalised to DMSO and pLKO and represent the mean values (±SD) from quadruplicate cultures, and were averaged from two independent experiments for each cell line. Statistically significant differences between control and inhibitor treatments are presented as **p < 0.01, ***p < 0.001. Right, CK2 stable silencing and CDC37 phosphorylation were evaluated by immunoblotting in GIST430 15 days after *CK2 shRNA* transduction, in the presence of puromycin selection.

Additive effects of combined KIT and CK2 inhibition were demonstrated by immunoblot, viability, cell cycle, apoptosis and colony-formation assays. KIT and CK2 signalling were evaluated by immunoblots in GIST-T1, GIST882, GIST430/654 and GIST48 after 6 h of treatment with imatinib and/or CX4945 in serum-free conditions (Fig. 3a). As described previously,^15^ imatinib treatment inactivated KIT and downstream signalling intermediates AKT, MAPK and S6 in imatinib-sensitive GIST-T1 and GIST882, whereas imatinib treatment partially decreased levels of p-KIT, p-AKT, p-MAPK and p-S6 in GIST48, and p-AKT and p-S6 expression in GIST430/654. Combination inhibition of KIT and CK2 by imatinib and CX4945 resulted in greater inactivation of KIT/PI3K/AKT/mTOR signalling in GIST882, GIST430/654 and GIST48, and MAPK in GIST48, compared with imatinib or CX4945 treatment alone. An additive effect was not observed in GIST-T1, for which imatinib and CX4945 alone provided near-maximal inhibition (Fig. 3a). Quantitations of KIT signalling expression are shown in Fig. 3a. CX4945 treatment had little impact on CK2 expression in GIST-T1 and GIST430/654 (Supplementary Fig. 2).

Additive effects on cell viability/cytotoxicity and colony-formation inhibition were observed after combined inhibition of KIT and CK2 in all four GIST cell lines (Fig. 3b–d; Supplementary Fig. 3A, B). KIT inhibition by imatinib at day 6 resulted in a 30–90% reduction in GIST cell viability, as compared with the DMSO control (Fig. 3b). Treatment with 2.5 and 5 µM CX4945 at day 6 showed minimal impact on viability in GIST882, GIST430/654 and GIST48, and resulted in a 60 and 90% reduction in GIST-T1 cell viability, respectively (Fig. 3b). However, combination treatment with imatinib and CX4945 (5 µM) resulted in 60 and 75% reduction in viability for GIST430/654 and GIST48 cell lines, respectively, whereas combination treatment in GIST-T1 and GIST882 cells resulted in 97 and 55% viability reduction (Fig. 3b). Anti-proliferative effects for CX4945, IM and combination of CX4945 with IM were further evaluated in all four GIST cell lines (Supplementary Fig. 3A, B). CX4945 IC50s in GIST-T1, GIST882, GIST430/654 and GIST48 were 6.8, 16.4, 4.7 and 11 µM, respectively, and IM IC50s in GIST-T1, GIST882, GIST430/654 and GIST48 were 0.039, 0.5, 0.66 and 0.09 µM, respectively. Combination treatment in GIST-T1, GIST882, GIST430/654 and GIST48 cells resulted in 77, 74, 65 and 77% viability reduction, respectively (Supplementary Fig. 3A, B).

Colonies of GIST cells treated with imatinib (GIST430/654 and GIST-T1) or CX4945 (GIST-T1) were fewer and smaller in size when compared with DMSO-treated cells, but not CX4945-treated GIST430/654 cells (Fig. 3c, d). Combination inhibition further decreased the size and number of colonies in GIST-T1 and GIST430/654, as compared with either intervention alone (Fig. 3c, d). Relative to DMSO, colony formation decreased by 35 (GIST-T1) and 15% (GIST430/654) in cells treated with CX4945, by 50% in cells treated with imatinib and by 90 (GIST-T1) and 80% (GIST430/654) in cells treated with CX4945 and imatinib (Fig. 3c, d). These results were statistically significant (*p* < 0.01).

GIST-T1, GIST882, GIST430/654 and GIST48 cells were further evaluated by cell cycle assays (Fig. 3e). Combination treatment with CX4945 and imatinib induced greater apoptosis in GIST430/654 and GIST48 than each intervention alone: nuclear fragmentation was demonstrated in 12.3 and 50.1% of cells treated with imatinib, in 10.3 and 7.8% of cells treated with CX4945 and in 18.2 and 54.4% of those treated with both imatinib and CX4945 (Fig. 3e). As described previously,^15^ cell cycle analysis also demonstrated apoptosis in imatinib-sensitive GIST-T1 and GIST882 cells after imatinib treatment, but not after CX4945 treatment: nuclear fragmentation was observed in 17.7 and 15.3% of cells treated with imatinib, in comparison with 2.2 and 0.9% of cells treated with DMSO control. Induction of apoptosis in GIST-T1 and GIST882 cells after combination of imatinib and CX4945 treatment was greater than after KIT suppression alone, with nuclear fragmentation observed in 35.3 and 48.1% of cells treated with imatinib and CX4945 (Fig. 3e).

In apoptotic assays, treatment of GIST430/654 and GIST48 with both imatinib and CX4945 for 72 h showed a greater increase in apoptotic cells, as compared with each intervention alone (37.4% for GIST430/654, in contrast with 31% for imatinib and 20.5% for CX4945; 44.1% for GIST48, in contrast with 39.5% for imatinib, and 3.4% for CX4945) (Fig. 3f; Supplementary Table S1). Levels of apoptosis in GIST-T1 and GIST882 cells after combination of imatinib and CX4945 treatment (45 and 63.8%, respectively) were greater than after imatinib (14.3 and 14.9%, respectively) or CX4945 (6.8 and 13.3%) treatment alone (Fig. 3f; Supplementary Table S1).

Finally, additive effects on cell viability were further demonstrated after combination of CK2 shRNA knockdown and KIT inhibition (Fig. 3g). CK2 shRNA knockdown inhibited p-CDC37 expression; co-targeting of CK2 and KIT by shRNA and imatinib resulted in greater viability reduction in GIST430, as compared with CK2 shRNA or imatinib treatment alone.

### Additive anti-invasive effects via dual targeting of CK2 and KIT in GIST

**Fig. 4:**
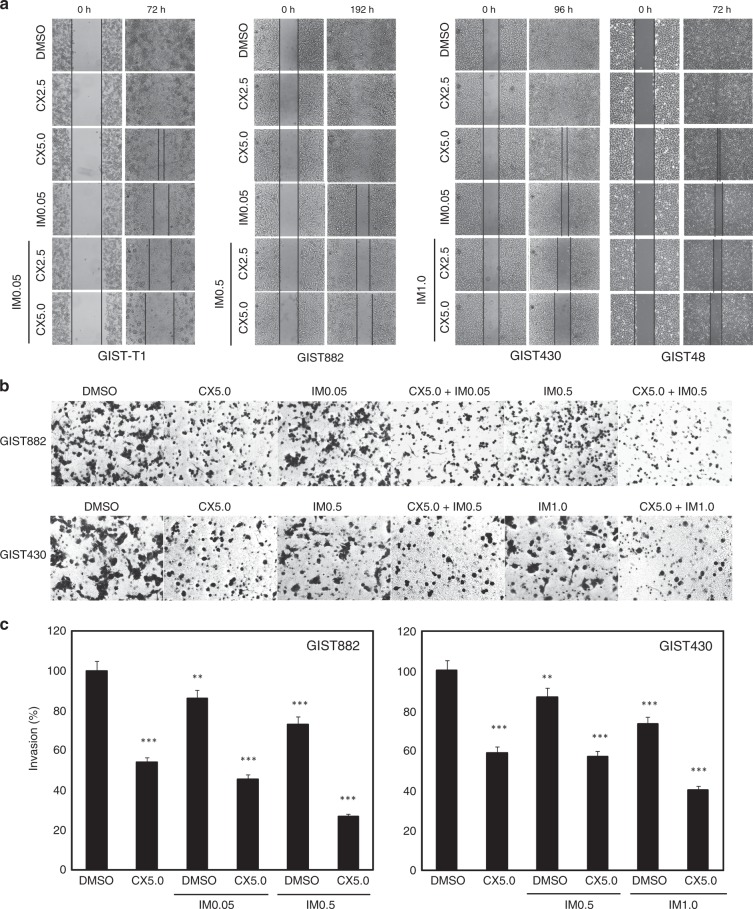
Combination treatment with imatinib (IM) and CX4945 (CX) inhibits migration and invasion in GIST cell lines (GIST-T1, GIST882, GIST430/654 and GIST48). In vitro wounding assays (**a**) and transwell migration assays (**b**) show that combination treatment with IM and CX more effectively inhibits migration and invasion of GIST cell lines than either intervention alone. Wound-healing and transwell experiments were performed in triplicate. **c** Quantitation of GIST cell invasiveness after treatment with IM and CX4945 for 72 h. Statistically significant differences between untreated control and inhibitor treatments are presented as **p < 0.01, ***p < 0.001.

Assays were performed in GIST-T1, GIST882, GIST430/654 and GIST48 to evaluate the effects of CK2 and KIT inhibition on GIST cell migration and invasion. Wound-healing assays in GIST-T1, GIST882, GIST430/654 and GIST48 cells demonstrated that combination treatment with imatinib and CX4945 impaired wound closure at 3, 8 and 4 days to a greater extent than either intervention alone, whereas complete wound closure was seen in DMSO-treated control cells (Fig. 4a). Matrigel assays demonstrated similar results, with 40% inhibition of invasiveness after CX4945 treatment, and 60% inhibition after combination treatment with imatinib in imatinib-resistant GIST430/654 cells, as compared with the DMSO control (Fig. 4b, c). As described previously,^15^ in imatinib-sensitive GIST882, treatment with imatinib alone led to 25% of invasion inhibition. CX4945 alone inhibited 45% invasiveness, while treatment with a combination of imatinib and CX4945 led to the greatest inhibition of 75% (Fig. 4b, c).

### CK2 inhibition blocks the interaction of KIT:HSP90:CDC37 in GIST

**Fig. 5:**
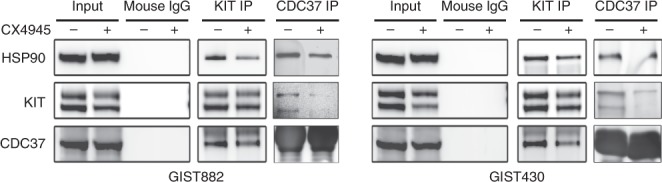
CX4945 treatment blocked the interactions of KIT–HSP90–CDC37. The KIT–HSP90–CDC37 complexes were evaluated after treatment with CX4945 (5 μM) for 6 h by KIT or CDC37 immunoprecipitation, followed by HSP90, KIT and CDC37 immunoblotting. The normal mouse IgG immunoprecipitation is a negative control. Immunoblotting and immunoprecipitation experiments were performed from two independent experiments for each cell line.

To evaluate the effects of CK2 inhibition on KIT:HSP90:CDC37 interactions, KIT and CDC37 immunoprecipitations (co-IP) and HSP90, KIT and CDC37 immunoblotting were performed in GIST882 and GIST430/654 after treatment with DMOS or CX4945 for 6 h (Fig. 5). Normal mouse IgG control co-IP did not show bands (KIT/HSP90/CDC37) at the positions of interest. As compared with the DMSO control treatment, CX4945 treatment decreased the intensity of the HSP90 and the CDC37 bands in KIT co-IPs, and the HSP90 and KIT bands in the CDC37 co-IPs in GIST882 and GIST430/654 (Fig. 5). Taken together, these results indicate that CK2 inhibition by CX4945 leads to decreased interactions of KIT, HSP90 and CDC37 in GIST cells.

## Discussion

Most GISTs harbour KIT or PDGFRA kinase gain-of-function mutations, and therefore respond clinically to imatinib and other TKI therapies. However, clinical progression subsequently results from various TKI-resistant mechanisms, typically secondary mutations in the KIT kinase domain, which can be heterogeneous between and within GIST metastases in a given patient. Preclinical validations have shown compelling responses to HSP90 inhibition in GIST, in vitro and in vivo: KIT oncoproteins are rapidly degraded after treatment with a variety of HSP90 inhibitors, and result in anti-proliferative and pro-apoptotic consequences.^29,30^ Although constitutively active KIT oncoproteins require chaperone HSP90 and are potently inactivated by HSP90 inhibitors, clinical trials in GIST patients still are underway because of toxicity resulting from concomitant inactivation of various other HSP90 client proteins, beyond KIT and PDGFRA.

In our previous work, we identified the HSP90 cofactor, CDC37, as one of the top GIST-specific essential genes by parallel genome-scale short-hairpin RNA (shRNA)-mediated gene knockdowns in GIST-T1 and GIST882.^35^ Subsequent functional evaluations highlighted that CDC37 is a crucial regulator of KIT oncoproteins in both imatinib-sensitive and imatinib-resistant GIST, and thus represents a promising strategy for inactivating the heterogeneous mutant KIT oncoproteins in TKI-resistant GIST patients. Therefore, effective inhibition of CDC37 would provide a selective indirect mechanism to inactivate KIT in GIST, especially in imatinib-resistant GISTs. One strategy for selective CDC37 targeting might result from pharmacologic dysregulation of phosphorylation at CDC37 Ser 13, which is mediated by CK2. CDC37 phosphorylation is a requisite for CDC37 recruitment of kinase clients to the HSP90 complex.^46^ Therefore, targeting CK2 might inhibit KIT-directed HSP90 functions in GIST, and co-targeting of CK2 and KIT could exhibit greater anti-proliferative and pro-apoptotic effects in GISTs than either intervention alone.

In our initial studies, we have evaluated the expression of CK2 in GIST cell lines and primary frozen tumours (Fig. 1a, b). As compared with other cancer cell lines and adjacent non-neoplastic tissues, overexpression of CK2 and phosphorylation of CDC37 was demonstrated in GIST cell lines and biopsies, indicating that CK2 could be an important functional regulator in GIST. Interestingly, the only non-GIST cell line with CK2 expression comparable with that of GIST was an RTK-driven NSCLC cell line, PC-9, which harbours an EGFR-activating mutation. This finding suggests that the anticancer effects of CK2 inhibition presented herein may apply more broadly to RTK-driven tumours. A single GIST biopsy, P5, did not show detectable expression of CK2, p-CDC37, p-KIT and KIT (Fig. 1b); although genomic analysis of this sample from a very low-risk GIST confirmed a *KIT* V560D mutation, the immunoblotting results raise the possibility of a biopsy sample with too low neoplastic component. Overall, the expression level of CK2 parallels expression and activation of KIT in GIST (Fig. 1b), suggesting a correlation between CK2 and KIT expression and activation.

At the biochemical level, CK2 inhibition by CX4945 resulted in dephosphorylation of CDC37 and partially inactivated KIT and PI3K/AKT/mTOR signalling in GIST-T1, GIST882 and GIST48 (Fig. 2). These data suggest that targeting CK2 inhibited KIT signalling in GIST, via regulation of CDC37 phosphorylation. However, CX4945 treatment did not decrease dephosphorylation of CDC37 and KIT/PI3K/AKT/mTOR signalling in GIST430/654 cells (Fig. 2), which harbours heterozygous KIT mutations and retains wild-type KIT molecules that may be more stable than mutant oncoproteins and less dependent on the HSP90 chaperone machinery; this interpretation is supported by the differential profile observed in these imatinib-resistant GIST cell lines upon HSP90 inhibition by 17-AAG.^29^ These data suggest that CK2 regulates GIST cell proliferation via CDC37/HSP90/KIT complexes, which also demonstrated that the interaction of CDC37:HSP90:KIT was blocked by CK2 inhibition (Fig. 5). Further investigation showed the additive effects in imatinib-sensitive GIST-T1 and GIST882, and imatinib-resistant GIST430/654 and GIST48 via coordinated targeting of KIT (with imatinib) and CK2 (with CX4945/shRNA), evidencing inactivation of KIT and downstream intermediates AKT, mTOR and MAPK, cell viability, colony-formation assays, apoptosis, cell cycle analysis, wound healing and invasiveness (Figs. 3, 4; Supplementary Fig. 3). In these experiments, the combination of CX4945 and imatinib significantly induced apoptosis, and resulted in decreased phosphorylation of KIT and AKT; the additive reduction of CDC37, MAPK and S6 phosphorylation was limited (Fig. 3a), given that CX4945 alone achieves almost maximal inhibition at 5 µM; in this context, the possibility of off-target effects (toxicity) contributing to apoptosis induction cannot be completely excluded (Fig. 3f). The present data suggest that CK2 inhibition results in reduced CDC37 phosphorylation and decreased interactions of CDC37 and HSP90, resulting in inactivation of KIT and downstream intermediates. These novel findings suggest that CK2 inhibition enhances imatinib anti-proliferative effects in GISTs, especially in imatinib-resistant GISTs.

In conclusion, CK2 is overexpressed in GIST cells and plays a critical role in CDC37 regulation, modulating HSP90 function and ultimately impacting KIT activity in GIST. Based on the evidence presented in this report, we believe that CK2/CDC37/KIT signalling is essential for GIST proliferation and survival, and that dual targeting of KIT and CK2 warrants evaluation as a novel therapeutic strategy in imatinib-sensitive and imatinib-resistant GIST.

## Supplementary information

Supplemental information

## Data Availability

All data generated or analysed during this study are included in this published article.

## References

[1] Hirota S, Isozaki K, Moriyama Y, Hashimoto K, Nishida T, Ishiguro S (1998). Gain-of-function mutations of c-kit in human gastrointestinal stromal tumors. Science.

[2] Heinrich MC, Corless CL, Duensing A, McGreevey L, Chen CJ, Joseph N (2003). PDGFRA activating mutations in gastrointestinal stromal tumors. Science.

[3] Corless CL, Fletcher JA, Heinrich MC (2004). Biology of gastrointestinal stromal tumors. J. Clin. Oncol..

[4] Heinrich MC, Corless CL, Blanke CD, Demetri GD, Joensuu H, Roberts PJ (2006). Molecular correlates of imatinib resistance in gastrointestinal stromal tumors. J. Clin. Oncol..

[5] Liegl-Atzwanger B, Fletcher JA, Fletcher CD (2010). Gastrointestinal stromal tumors. Virchows Arch..

[6] Tuveson DA, Willis NA, Jacks T, Griffin JD, Singer S, Fletcher CD (2001). STI571 inactivation of the gastrointestinal stromal tumor c-KIT oncoprotein: biological and clinical implications. Oncogene.

[7] Demetri GD, von Mehren M, Blanke CD, Van den Abbeele AD, Eisenberg B, Roberts PJ (2002). Efficacy and safety of imatinib mesylate in advanced gastrointestinal stromal tumors. N. Engl. J. Med..

[8] Heinrich MC, Maki RG, Corless CL, Antonescu CR, Harlow A, Griffith D (2008). Primary and secondary kinase genotypes correlate with the biological and clinical activity of sunitinib in imatinib-resistant gastrointestinal stromal tumor. J. Clin. Oncol..

[9] Fletcher JA (2016). KIT oncogenic mutations: biologic insights, therapeutic advances, and future directions. Cancer Res..

[10] Blay JY, Bonvalot S, Casali P, Choi H, Debiec-Richter M, Dei Tos AP (2005). Consensus meeting for the management of gastrointestinal stromal tumors. Report of the GIST Consensus Conference of 20-21 March 2004, under the auspices of ESMO. Ann. Oncol..

[11] Demetri, G. D., Heinrich, M. C., Fletcher, J. A., Fletcher, C. D. Van den Abbeele, A. D., Corless, C. L. et al. Molecular target modulation, imaging, and clinical evaluation of gastrointestinal stromal tumor patients treated with sunitinib malate after imatinib failure. *Clin. Cancer Res.* **15**, 5902–5909 (2009). 10.1158/1078-0432.CCR-09-0482 PMC3417101 19737946

[12] Demetri GD, Reichardt P, Kang YK, Blay JY, Rutkowski P, Gelderblom H (2013). Efficacy and safety of regorafenib for advanced gastrointestinal stromal tumours after failure of imatinib and sunitinib (GRID): an international, multicentre, randomised, placebo-controlled, phase 3 trial. Lancet..

[13] Essat M, Cooper K (2011). Imatinib as adjuvant therapy for gastrointestinal stromal tumors: a systematic review. Int. J. Cancer.

[14] Liegl B, Kepten I, Le C, Zhu M, Demetri GD, Heinrich MC (2008). Heterogeneity of kinase inhibitor resistance mechanisms in GIST. J. Pathol..

[15] Chen W, Kuang Y, Qiu HB, Cao Z, Tu Y, Sheng Q (2017). Dual targeting of insulin receptor and KIT in imatinib-resistant gastrointestinal stromal tumors. Cancer Res..

[16] Mahadevan D, Theiss N, Morales C, Stejskal AE, Cooke LS, Zhu M (2015). Novel receptor tyrosine kinase targeted combination therapies for imatinib-resistant gastrointestinal stromal tumors (GIST). Oncotarget.

[17] Tu Y, Zuo R, Ni N, Eilers G, Wu D, Pei Y (2018). Activated tyrosine kinases in gastrointestinal stromal tumor with loss of KIT oncoprotein expression. Cell Cycle.

[18] Mahadevan D, Cooke L, Riley C, Swart R, Simons B, Della Croce K (2007). A novel tyrosine kinase switch is a mechanism of imatinib resistance in gastrointestinal stromal tumors. Oncogene.

[19] Ou WB, Ni N, Zuo R, Zhuang W, Zhu M, Kyriazoglou A (2019). Cyclin D1 is a mediator of gastrointestinal stromal tumor KIT-independence. Oncogene.

[20] de Thonel A, Hazoumé A, Kochin V, Isoniemi K, Jego G, Fourmaux E (2014). Regulation of the proapoptotic functions of prostate apoptosis response-4 (Par-4) by casein kinase 2 in prostate cancer cells. Cell Death Dis..

[21] Niechi I, Silva E, Cabello P, Huerta H, Carrasco V, Villar P (2015). Colon cancer cell invasion is promoted by protein kinase CK2 through increase of endothelin-converting enzyme-1c protein stability. Oncotarget.

[22] Kren BT, Unger GM, Abedin MJ, Vogel RI, Henzler CM, Ahmed K (2015). Preclinical evaluation of cyclin dependent kinase 11 and casein kinase 2 survival kinases as RNA interference targets for triple negative breast cancer therapy. Breast Cancer Res..

[23] Liu Y, Amin EB, Mayo MW, Chudgar NP, Bucciarelli PR, Kadota K (2016). CK2alpha’ drives lung cancer metastasis by targeting BRMS1 nuclear export and degradation. Cancer Res..

[24] Siddiqui-Jain A, Drygin D, Streiner N, Chua P, Pierre F, O’Brien SE (2010). CX-4945, an orally bioavailable selective inhibitor of protein kinase CK2, inhibits prosurvival and angiogenic signaling and exhibits antitumor efficacy. Cancer Res..

[25] Pierre F, Chua PC, O’Brien SE, Siddiqui-Jain A, Bourbon P, Haddach M (2011). Pre-clinical characterization of CX-4945, a potent and selective small molecule inhibitor of CK2 for the treatment of cancer. Mol. Cell Biochem..

[26] Rabalski AJ, Gyenis L, Litchfield DW (2016). Molecular pathways: emergence of protein kinase CK2 (CSNK2) as a potential target to inhibit survival and DNA damage response and repair pathways in cancer cells. Clin. Cancer Res..

[27] Siddiqui-Jain A, Bliesath J, Macalino D, Omori M, Huser N, Streiner N (2012). CK2 inhibitor CX-4945 suppresses DNA repair response triggered by DNA-targeted anticancer drugs and augments efficacy: mechanistic rationale for drug combination therapy. Mol. Cancer Ther..

[28] Yang B, Yao J, Li B, Shao G, Cui Y (2017). Inhibition of protein kinase CK2 sensitizes non-small cell lung cancer cells to cisplatin via upregulation of PML. Mol. Cell Biochem..

[29] Bauer S, Yu LK, Demetri GD, Fletcher JA (2006). Heat shock protein 90 inhibition in imatinib-resistant gastrointestinal stromal tumor. Cancer Res..

[30] Smyth T, Van LT, Curry JE, Rodriguez-Lopez AM, Wozniak A, Zhu M (2012). The HSP90 inhibitor, AT13387, is effective against imatinib-sensitive and -resistant gastrointestinal stromal tumor models. Mol. Cancer Ther..

[31] Shapiro GI, Kwak E, Dezube BJ, Yule M, Ayrton J, Lyons J (2015). First-in-human phase I dose escalation study of a second-generation non-ansamycin HSP90 inhibitor, AT13387, in patients with advanced solid tumors. Clin. Cancer Res..

[32] Wagner AJ, Agulnik M, Heinrich MC, Mahadevan D, Riedel RF, von Mehren M (2016). Dose-escalation study of a second-generation non-ansamycin HSP90 inhibitor, onalespib (AT13387), in combination with imatinib in patients with metastatic gastrointestinal stromal tumour. Eur. J. Cancer.

[33] Bendell JC, Bauer TM, Lamar R, Joseph M, Penley W, Thompson DS (2016). A phase 2 study of the Hsp90 inhibitor AUY922 as treatment for patients with refractory gastrointestinal stromal tumors. Cancer Invest..

[34] Eckl JM, Scherr MJ, Freiburger L, Daake MA, Sattler M, Richter K (2015). Hsp90.Cdc37 complexes with protein kinases form cooperatively with multiple distinct interaction sites. J. Biol. Chem..

[35] Marino-Enriquez A, Ou WB, Cowley G, Luo B, Jonker AH, Mayeda M (2014). Genome-wide functional screening identifies CDC37 as a crucial HSP90-cofactor for KIT oncogenic expression in gastrointestinal stromal tumors. Oncogene.

[36] Miyata Y, Nishida E (2004). Supervision of multiple signaling protein kinases by the CK2-Cdc37 couple, a possible novel cancer therapeutic target. Ann. N. Y. Acad. Sci..

[37] Miyata Y, Nishida E (2005). CK2 binds, phosphorylates, and regulates its pivotal substrate Cdc37, an Hsp90-cochaperone. Mol. Cell Biochem..

[38] Miyata Y, Nishida E (2007). Analysis of the CK2-dependent phosphorylation of serine 13 in Cdc37 using a phospho-specific antibody and phospho-affinity gel electrophoresis. FEBS J..

[39] Lux ML, Rubin BP, Biase TL, Chen CJ, Maclure T, Demetri G (2000). KIT extracellular and kinase domain mutations in gastrointestinal stromal tumors. Am. J. Pathol..

[40] Ou WB, Zhu MJ, Demetri GD, Fletcher CD, Fletcher JA (2008). Protein kinase C-theta regulates KIT expression and proliferation in gastrointestinal stromal tumors. Oncogene.

[41] Rubin BP, Singer S, Tsao C, Duensing A, Lux ML, Ruiz R (2001). KIT activation is a ubiquitous feature of gastrointestinal stromal tumors. Cancer Res..

[42] Elangovan S, Pathania R, Ramachandran S, Ananth S, Padia RN, Lan L (2014). The niacin/butyrate receptor GPR109A suppresses mammary tumorigenesis by inhibiting cell survival. Cancer Res..

[43] Shaw RJ, Paez JG, Curto M, Yaktine A, Pruitt WM, Saotome I (2001). The Nf2 tumor suppressor, merlin, functions in Rac-dependent signaling. Dev. Cell.

[44] Yang MH, Chang SY, Chiou SH, Liu CJ, Chi CW, Chen PM (2007). Overexpression of NBS1 induces epithelial-mesenchymal transition and co-expression of NBS1 and Snail predicts metastasis of head and neck cancer. Oncogene.

[45] Miyata Y, Nishida E (2008). Evaluating CK2 activity with the antibody specific for the CK2-phosphorylated form of a kinase-targeting cochaperone Cdc37. Mol. Cell Biochem..

[46] Xu W, Mollapour M, Prodromou C, Wang S, Scroggins BT, Palchick Z (2012). Dynamic tyrosine phosphorylation modulates cycling of the HSP90-P50(CDC37)-AHA1 chaperone machine. Mol. Cell.

