# Supplementary material for: Coordinated targeting of CK2 and KIT in gastrointestinal stromal tumours
Source: Br J Cancer. 2019 Nov 28;122(3):372–81. doi: 10.1038/s41416-019-0657-5 (PMC7000686; doi:10.1038/s41416-019-0657-5)
Supplement: Supplementary file 1 — Supplemental information [file 41416_2019_657_MOESM1_ESM.pdf]

Supplemental Table 1. Apoptosis evaluation (%) in GIST cell lines after treatment with CX4945 and imatinib.

Cell lines / inhibitors	GIST-T1			GIST882			GIST430			GIST48		
	Early	late	dead	early	late	dead	early	late	dead	early	late	dead
DMSO	0.3	**3.2**	3.8	0	**4.2**	7.9	0.7	**6.3**	18	0	**14.4**	0.2
CX	0	**3.4**	2.4	0	**4.6**	8.7	0.2	**7.1**	13.2	0.1	**3.2**	0.1
IM1	0	**3.4**	3.5	0	**4.1**	10.1	0	**7.2**	14.5	1.5	**13.7**	29.2
IM1+CX	0	**5.6**	11	0.3	**21**	38.4	0.2	**11**	22.2	1.0	**18.3**	26
IM2	0	**6.5**	7.8	0	**5.1**	9.8	0.1	**8.4**	22.5	1.6	**12.1**	25.8
IM2+CX	0	**30.5**	14.5	0.2	**22.6**	41	0	**12**	25.4	1.6	**16.3**	26.2

Notes: GIST cells in six-well plates were treated with CX4945 (5 μM) or imatinib (0.05 and 0.5 μM for GIST-T1 and GIST882; 0.5 and 1 μM for GIST430/654 and GIST48).

SFigure 1

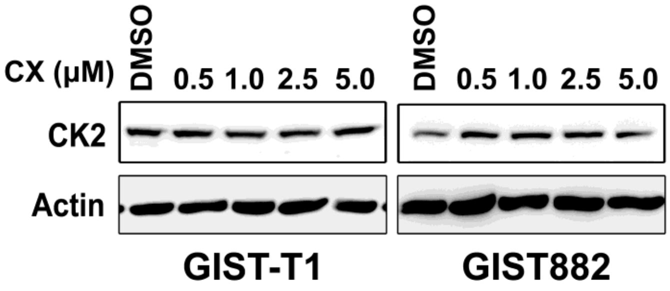

Supplemental Figure 1. Immunoblotting evaluation of CK2 expression in GIST-T1 and GIST882 cells after treatment with the CK2 inhibitor CX4945 (0.5, 1.0, 2.5, and 5 μM) for 6 h in serum-free medium. Actin stain is a loading control. CX4945 treatment had no impact on CK2 expression in GIST-T1 and GIST882. Immunoblotting experiments were performed from two independent replicates for each sample.

SFigure 2

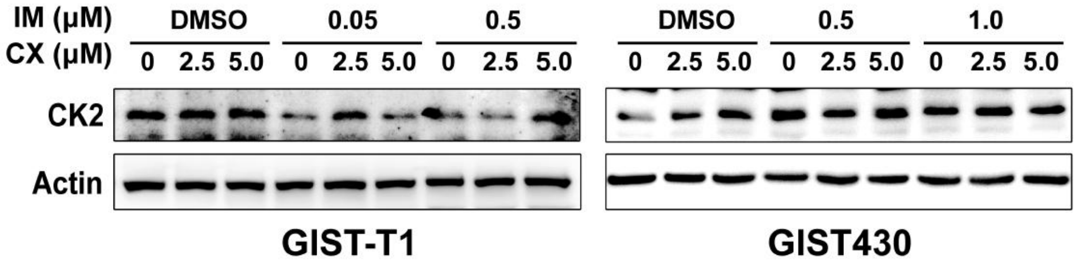

Supplemental Figure 2. CK2 expression was evaluated in GIST-T1 and GIST430/654 by immunoblotting 6 h after treatment with CX4945 (CX: 2.5 and 5 μM) and imatinib (IM: 0.05, 0.5 μM, and 1 μM). Actin stain is a loading control. CX4945 treatment had no impact on CK2 expression in both GIST cell lines. Immunoblotting experiments were performed from two independent replicates for each cell line.

SFigure 3

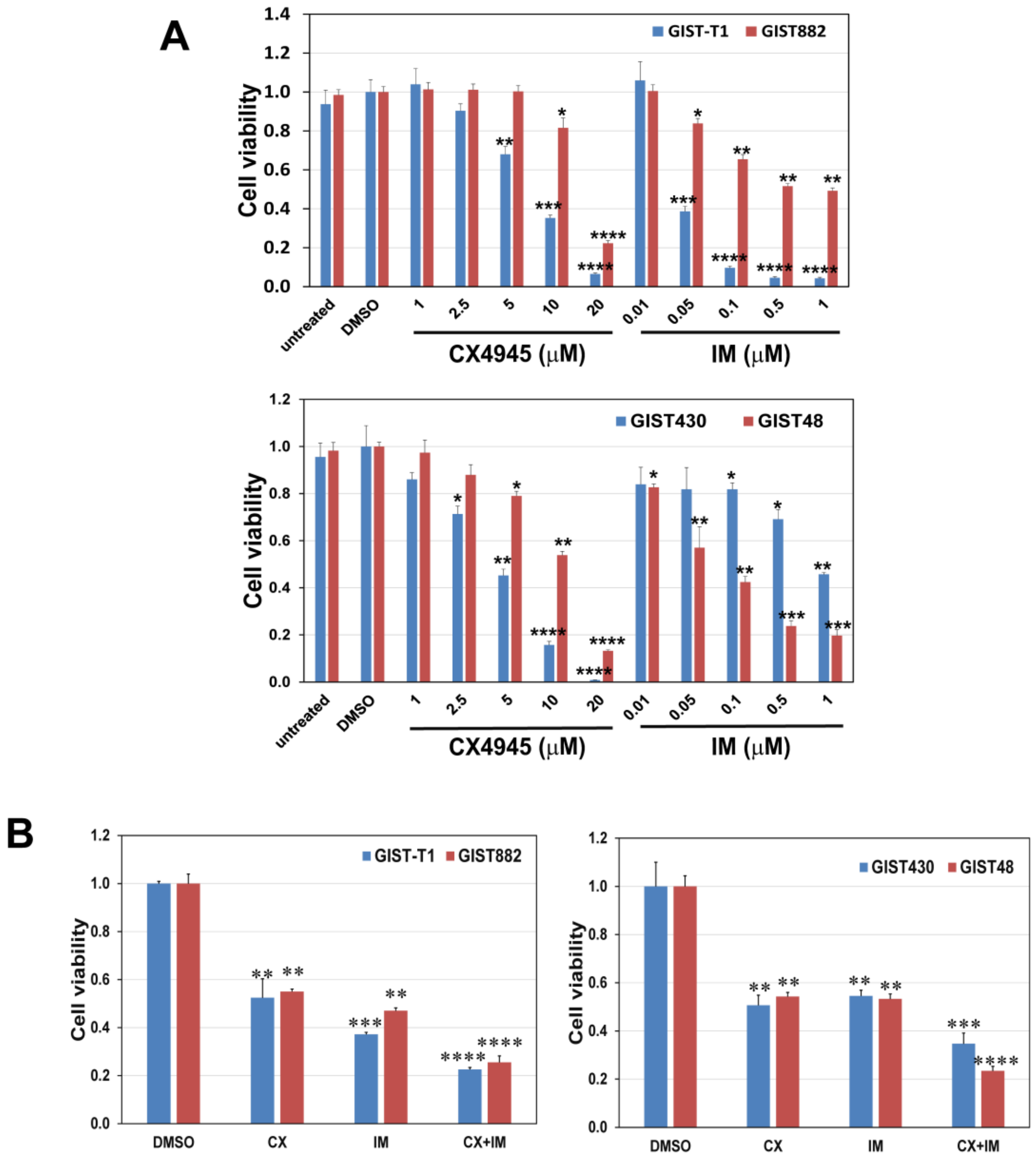

Supplemental Figure 3. Additive effects of coordinated inhibition of KIT and CK2 as demonstrated by cell viability (**A** and **B**), showing that combined inhibition of KIT and CK2 induced greater anti-proliferative effects than either intervention alone in GIST cell lines (GIST-T1, GIST882, GIST430/654, and GIST48). **A**) Cell viability was evaluated by a Cell-titer Glo® ATP-based luminescence assay in GIST cell lines

(GIST-T1, GIST882, GIST430/654, and GIST48), 6 days after treatment with CX4945 (1, 2.5, 5, 10, and 20 μM) and IM (0.01, 0.05, 0.1, 0.5, and 1 μM). Data are normalized to DMSO and represent the mean values (± s.d.) from quadruplicate wells, averaged from two independent experiments for each cell line. Statistically significant differences between untreated control and inhibitor treatments are presented as *$p < 0.05$, **$p < 0.01$, ***$p < 0.001$, ****$p < 0.0001$. **B**) Cell viability was evaluated by a Cell-titer Glo® ATP-based luminescence assay in GIST cell lines, 6 days after treatment with CX4945 (6.8 μM for GIST-T1, 16.4 μM for GIST882, 4.7 μM for GIST430/654, and 11 μM for GIST48) and IM (0.039 μM for GIST-T1, 0.5 μM for GIST882, 0.66 μM for GIST430/654, and 0.09 μM for GIST48). Data are normalized to DMSO and represent the mean values (± s.d.) from quadruplicate wells, averaged from two independent experiments for each cell line. Statistically significant differences between untreated control and inhibitor treatments are presented as **$p < 0.01$, ***$p < 0.001$, ****$p < 0.0001$.
